# Compilation of morphological and molecular data, a necessity for taxonomy: The case of *Hormogaster abbatissae* sp. n. (Annelida, Clitellata, Hormogastridae)

**DOI:** 10.3897/zookeys.242.3996

**Published:** 2012-11-15

**Authors:** Marta Novo, Rosa Fernández, Daniel Fernández Marchán, Mónica Gutiérrez, Darío J. Díaz Cosín

**Affiliations:** 1 Departamento de Zoología y Antropología Física, Facultad de Biología, Universidad Complutense de Madrid, C/ José Antonio Nováis 2, 28040, Madrid, Spain; 2 Cardiff School of Biosciences, Cardiff University, BIOSI 1, Museum Avenue, Cardiff CF10 3TL, UK; 3 Museum of Comparative Zoology, Department of Organismic and Evolutionary Biology, Harvard University, 26 Oxford Street, Cambridge, MA 02138, USA

**Keywords:** Species description, earthworm, morphological characters, molecular data, integrative taxonomy, homoplasy

## Abstract

Conflict among data sources can be frequent in evolutionary biology, especially in cases where one character set poses limitations to resolution. Earthworm taxonomy, for example, remains a challenge because of the limited number of morphological characters taxonomically valuable. An explanation to this may be morphological convergence due to adaptation to a homogeneous habitat, resulting in high degrees of homoplasy. This sometimes impedes clear morphological diagnosis of species. Combination of morphology with molecular techniques has recently aided taxonomy in many groups difficult to delimit morphologically. Here we apply an integrative approach by combining morphological and molecular data, including also some ecological features, to describe a new earthworm species in the family Hormogastridae, *Hormogaster abbatissae* **sp. n.**, collected in Sant Joan de les Abadesses (Girona, Spain). Its anatomical and morphological characters are discussed in relation to the most similar Hormogastridae species, which are not the closest species in a phylogenetic analysis of molecular data. Species delimitation using the GMYC method and genetic divergences with the closest species are also considered. The information supplied by the morphological and molecular sources is contradictory, and thus we discuss issues with species delimitation in other similar situations. Decisions should be based on a profound knowledge of the morphology of the studied group but results from molecular analyses should also be considered.

## Introduction

Traditional methods for identifying earthworm species and their phylogenetic relationships (i.e., the study of their morpho-anatomical features) have been limited by high levels of homoplasy. The structural simplicity of earthworms, the low degree of variability and the overlap of diagnostic characters among species, the absence of a fossil record and their adaptation to life in the soil, are the principal factors responsible for the difficulties in recognizing species. DNA sequence data has however facilitated the distinction of closely related species and may be the solution to understanding the true level of biodiversity within morphologically-difficult groups, such as earthworms.

Some degree of controversy has arisen on how to describe and delimit species, but discrete morphological features remain the most used criterion. Others are in favour of molecular-based descriptions (e.g., Cook et al. 2010) who justify species descriptions based solely on DNA sequences, even ignoring morphological data, but they also recognize that in cases with incomplete molecular databases – as for most taxa –, this alternative alone is not viable. Species descriptions including both morphological and DNA-based data are imperative for a more universal taxonomy. There are many authors in favor of this integrative taxonomy, consisting in a multidisciplinary approach including morphological, molecular, ecological and geographical data. This type of approach can include complex procedures therefore using multi-gene genetic distances, analyses such as General Mixed Yule-Coalescent (GMYC) models or Automatic Barcode Gap Discovery (ABGD) analyses and weighting of the established hypotheses with complementary data such as morphological, geographical or ecological (see Puillandre et al. 2012 and included references).

Hormogastridae includes middle to large-sized earthworms, currently comprising 27-29 species and subspecies that are exclusively distributed in the western Mediterranean (Díaz Cosín et al. 1989, Cobolli Sbordoni et al. 1992, Blakemore 2004, 2008), where they play a very important ecological role (Bouché 1972). The highest abundance of species seems to be located in the NE Iberian Peninsula, where more than a dozen species have been described.

The taxonomy of this group, as in other earthworm families, has been based until now solely on morphological features. The first species described are *Hormogaster redii* Rosa, 1887 and *Hormogaster pretiosa* Michaelsen, 1899. Subsequently, other species were added to the group by different authors, including Cognetti (1914), Zicsi (1970), Bouché (1970), Álvarez (1971, 1977), Díaz Cosín et al. (1989), and Rota (1993) but most were described by Qiu and Bouché (1998), including eleven new species from Spain presenting very subtle morphological differences. The known species are grouped in four genera, *Hormogaster* Rosa, 1887 (22-24 species and subspecies), *Hemigastrodrilus* Bouché, 1970 (one or two subspecies), *Vignysa* Bouché, 1970 (two species) and *Xana* Díaz Cosín et al., 1989 (one species).

Omodeo (1956) provided the first revision of the family in 1956, and later on Omodeo and Rota (2008) presented additional considerations on their evolution in an article including different Mediterranean areas. Cobolli Sbordoni et al. (1992) provided the first phylogenetic hypothesis of the family using allozymes, but that seminal work lacks a comprehensive sampling in NE Spain, where most of the hormogastrid diversity concentrates (Qiu and Bouché 1998). More recently, Novo et al. (2009, 2010) used DNA sequence data from multiple markers to detect cryptic diversity within *Hormogaster elisae* in the central area of the Iberian Peninsula. These studies highlight, among other aspects, the morphological stasis present in this group, whose anatomy seems to have adapted to the dry soils of this region.

During a collecting trip for the phylogenetic study of Novo et al. (2011), 22 hormogastrid specimens were collected near Sant Joan de les Abadesses (Girona, Spain). The specimens were assigned to *Hormogaster*, but were thought to represent a new species here described as *Hormogaster abbatissae* sp. n. Its description, including its relationship to other the closely related hormogastrid species are the initial objectives of this paper. The description we provide is complemented with a molecular analysis of different genes in the closest species, GMYC species delimitation and ecological data. This study resembles the first example to describe a new earthworm species by combining all these different data sources (Blakemore and Kupriyanova 2010, see also Blakemore 2010, Blakemore et al. 2010 and for lumbricids Blakemore and Grygier 2011 and Blakemore 2012) and also other studies on different taxa (e.g. Chullasorn et al. 2011, Heethoff et al. 2011, Hart et al. 2012). However, this is the first work to do so for an hormogastrid. Contradictory results between morphological and molecular data are found, and whether a particular data set should be favored over the remaining sources is discussed.

We expect that this example, combining molecular and morphological data and including ecological features, goes beyond the specific interest of a new earthworm species description and could be applied to other groups with comparable taxonomic problems.

## Material and methods

Specimens were collected by hand and fixed in the field in ca. 96% EtOH, with subsequent alcohol changes. Once in the laboratory, specimens were preserved at -20 °C.

The studied material includes 22 specimens (five mature specimens, four semi-mature specimens with tubercula pubertatis and/or clitellum draft and 13 immatures or fragments) collected between Ripoll and Sant Joan de les Abadesses, road C26, km 210 in a little forest near the Ter river (42°13'30.0"N, 2°14'57.5"E). Mean annual temperature is 14.3 °C and mean annual precipitation is 724 mm, as indicated by the nearest weather station (in the airport of Girona, 55km away: http://www.aemet.es/es/serviciosclimaticos/datosclimatologicos/valoresclimatologicos?l=0367&k=cat)

Specimens have been deposited in the Oligochaete Cryo collection of the *Departamento de Zoología y Antropología Física, Universidad Complutense de Madrid* (DZAF, UCM), Spain.

Specimens of nearly all other hormogastrid species were examined for comparison (list of specimens in Novo et al. 2011). Morphological characters include those features traditionally used for hormogastrids and other earthworms. Only the distantly related species *Hormogaster lleidana* Qiu and Bouché, 1998 and *Hormogaster multilamella* Qiu & Bouché, 1998 were not examined, and thus their information was limited to the published descriptions (Qiu and Bouché 1998). All the specimens are deposited in the earthworm criocollection of Complutense University of Madrid (DZAF, UCM).

Molecular data generation follow Novo et al. (2011, 2012). Phylogenetic inference and GMYC analyses discussed here are based on data published in those papers. Nine molecular regions of specimens SAN1, 2, 3, 4, 7, 8, 9, 10 were included: mitochondrial regions of cytochrome *c* oxidase subunit I (COI), 16S rRNA and tRNA Leu, Ala, and Ser, two nuclear ribosomal genes (complete 18S rRNA and a fragment of 28S rRNA) and two nuclear protein-encoding genes (histones H3 and H4). GeneBank accession numbers for the paragenetypes, following Chakrabarty (2010) for the mitochondrial markers, analyzed here are shown in Table 1.

We constructed networks with SplitsTree4 v.4.11.3 (Huson and Bryant 2006) for the mitochondrial genes (16S-tRNA, COI), including the phylogenetically closest species of *Hormogaster abbatissae* sp. n., in order to visualize in more detail the relationships and genetic distances among them. Default settings were used. We analysed 41 sequences of each gene including hormogastrids close to *Hormogaster abbatissae* sp. n.and *Hormogaster elisae* Álvarez, 1977 from Siguero and *Aporrectodea trapezoides* (Dugés 1828) as more distant outgroups (see Table 2). Uncorrected pairwise differences were calculated between these species with Arlequin 3.5 (Excoffier et al. 2005).

**Table 1.** Paragenetypes of *Hormogaster abbatissae* sp. n. with GenBank accession numbers. The holotype SAN 11 was not sequenced in order to preserve the specimen intact.

**Paragenetype**	**COI**	**16S-tRNA**
SAN1	JN209553	JN209358
SAN2	HQ621990	HQ621884
SAN3	JN209557	JN209360
SAN4	JN209555	JN209361
SAN7	JN209556	JN209362
SAN8	JN209559	JN209363
SAN9	JN209558	JN209364
SAN10	JN209554	JN209359

**Table 2.** Species represented in the network corresponding to the closest relatives of *Hormogaster abbatissae*, according to the phylogenetic study by Novo et al. (2011). More distantly related species appear in bold. GenBank accession numbers of the used sequences are shown for each gene.

**Species**	**Locality**	**Region, Country**	**Coordinates**	**N**	**COI**	**16S**
*Hormogaster sylvestris*	Montmajor	Barcelona, Spain	42°01'43.3"N, 001°42'43.7"E	2	JN209552, HQ621981	JN209286, HQ621874
*Hormogaster pretiosa nigra*	Quillan	Aude, France	42°52'48.8"N, 002°10'12.0"E	1	HQ621988	HQ621882
*Hormogaster catalaunensis*	El Brull	Barcelona, Spain	41°48'04.9"N, 002°20'51.6"E	1	HQ621973	HQ621866
*Hormogaster gallica*	Banyuls Sur Mer	Pyrénées-Orientales, France	42°28'08.0"N, 003°09'08.2"E	1	HQ621974	HQ621867
*Hormogaster arenicola*	Biosca	Lleida, Spain	41°51'04.6"N, 001°19'40.4"E	8	JN209493, JN209494, JN209495, JN209496, JN209497, JN209498, JN209499, HQ621972	JN209208, JN209209, JN209210, JN209211, JN209212, JN209213, JN209214, HQ621865
*Hormogaster riojana*	Alesanco	La Rioja, Spain	42°26'21.7"N, 002°50'18.4"W	10	JN209477, JN209478, JN209479, JN209480, JN209481, JN209482, JN209483, JN209484, JN209485, HQ621970	JN209196, JN209197, JN209198, JN209199, JN209200, JN209201, JN209202, JN209203, JN209204, HQ621862
*Hormogaster ireguana*	Torrecilla en Cameros	La Rioja, Spain	42°13'54.7"N, 002°37'35.2"W	8	JN209486, JN209487, JN209488, JN209489, JN209490, JN209491, JN209492, HQ621994	JN209394, JN209395, JN209396, JN209397, JN209398, JN209399, JN209400, HQ621888
*Hormogaster elisae*	Siguero	Madrid, Spain	41°11'06.1"N, 03°37'07.4"W	1	EF653894.1	GQ409710.1
*Hormogaster trapezoides*	San Román	Asturias, Spain	43°15'20.9"N, 005°05'10.3"W	1	JF313607	HQ621864

## Results

### Taxonomic results

**Phylum Annelida Lamarck, 1802**

**Subphyllum Clitellata Michaelsen, 1919**

**Class Oligochaeta Grube, 1850**

**Order Haplotaxida Michaelsen, 1900**

**Family Hormogastridae Michaelsen, 1900**

**Genus *Hormogaster* Rosa, 1887**

Type-species *Hormogaster redii* Rosa, 1887

#### Hormogaster abbatissae

Novo & Díaz Cosín sp. n.

urn:lsid:zoobank.org:act:6A388AC5-A2E4-4A32-9BA4-F0F1C5684EBE

http://species-id.net/wiki/Hormogaster_abbatissae

Hormogaster abbatissae Novo, 2010: 249 (eprints.ucm.es/12304/1/T32615.pdf) and Novo and Díaz Cosín, in press: (http://www.ucm.es/info/zoo/invertebrados/PDF/Novo%20et%20al%20%28en%20prensa%29%20When%20morphology%20and%20molecules%20clash.pdf) – nomina nuda superceded by current publication.

##### Material examined.

*Holotype*. Adult (Catalog # SAN11 DZAF, UCM), 42°13'30.0"N, 2°14'57.5"E, from a small patch of forest near the Ter river, road C26, Km 210, between Ripoll and Sant Joan de les Abadesses, Girona (Spain), leg. M. Novo, D. Díaz Cosín, R. Fernández, December 2006.

*Paratypes*. 21 specimens (Catalog # SAN1-10, 12-22 DZAF, UCM), same collecting data as holotype.

*Other material examined*. 16 *Hormogaster* species and several subspecies included in the study by Novo et al. (2011).

##### Morphological description.

**Figure 1.**
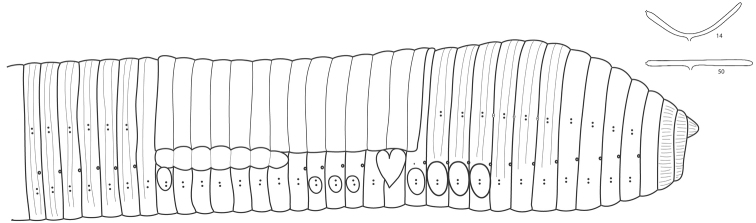
External morphology of *Hormogaster abbatissae*. An illustration of nephridial bladders in segments 14 and 50 is shown in the upper right corner.

*External morphology* (Figure 1). Length of the mature specimens: 103-130 mm. Maximum diameter (pre-clitellar, clitellar, post-clitellar): 8, 11, 9 mm. Number of segments: 239-270. Weight (fixed specimens): 3.45-4.98 g.

Colour: Anterior pink in live animals, with darker clitellum and grey-bluish posterior (Supplementary Figure S.1B). Specimens are grey-bluish when preserved in ethanol, with beige clitellum (Supplementary Figure S.1D).

Prostomium proepilobic 1/3. Segments 1 and 2 showing longitudinal lines. Chaetae closely paired, quite lateral, visible along the body as two faint blue lines; intersetal ratio at segment 50, *aa*: 50, *ab*: 1.5, *bc*: 9, *cd*: 1, *dd*: 52. Nephridial pores in a row, between chaetae *b* and *c*. Spermathecal pores at intersegments 8/9, 9/10 and 10/11, at the level of chaetae *cd*.

Male pores opening near the 15/16 as elongated fissures at the level of *ab*, showing heart-shaped porophores of variable developmental degree that can cover practically all width of the segment 15 and ½ of 16 in mature specimens. Female pores in 14 more or less at the same level as the male ones.

Clitellum saddle-shaped extending over 14,15–27. Tubercula pubertatis in (20) 21,22–26,27 appearing frequently as a continuous line in 21–27. Papillae with variable position, frequently situated at *ab* chaetae in segment 27, although more variable in other segments within the pre-clitellar and clitellar area.

*Internal anatomy*. Funnel-shaped and strongly thickened septa in 7/8, 8/9 and 9/10, also in 6/7 and 10/11, less thickened though. Last pair of hearts in 11. Three globular strongly muscular gizzards in 6, 7 and 8 of shining appearance. Not apparent Morren’s glands, although in transverse sections of the oesophagus at segments 10 to 14 some thickened blood vessels can be detected, but never the lamellae typically showed by this glands.

Lack of well-differentiated posterior gizzard, although the esophagus is a bit dilated at 15–16, but its wall is not especially muscular and its lumen does not exhibit a reinforcement similar to that in the anterior gizzards. In segments 17–25, 26, the gut shows folds in the wall of every segment, forming what has been called a stomach in some earthworms. Typhlosole begins in 20, 21 and presents 15 lamellae, being the two lateral ones very small that therefore could be unnoticed. Number of lamellae gradually decreases, showing three from segment 80 to 140–150, and one until 160–170 where the typhlosole ends. Therefore the last 70 to 100 segments lack the typhlosole.

Fraying testes and iridescent seminal funnels in 10 and 11. Two pairs of granular appearing seminal vesicles in 11 and 12 frequently showing black bodies. Ovaries and female funnels in 13; big ovarian receptacles in 14.

Three pairs of spermathecae in segments 8, 9 and 10 included into septa 8/9, 9/10 and 10/11 the ones in 8 being the smallest. Spermathecae with the appearance of flattened sacks, dish or flying saucer showing irregular borders inside the body wall under some of the muscular fascicles. They can be divided internally into interconnected lobes that in fact do not represent independent spermathecae but simple multicameral spermathecae that open to the exterior by a unique pore.

Anterior nephridial bladders V-shaped with widely open branches, being one of them shorter. They flatten towards the posterior section of the body, until the extent of showing appearance of an elongated sausage.

In some of the specimens, the sexual chaetae in 11 and 12 present well developed follicles that go into the body as a projection where various chaetae simultaneously appear.

##### Distribution.

Known only from its type locality.

##### Habitat.

Specimens were collected in a small forest patch dominated by *Populus alba*, *Acer pseudoplatanus* and *Rosa canina*, which develops in a slope at the edge of a meadow. The soil was covered with abundant leaf litter (Supplementary Figure S1. A), and it is characterized by 13.57% of coarse sand, 9.62% fine sand, 6.27% coarse silt, 32.37% fine silt, and 38.18% clay, constituting a clay loam soil, carbon (C): 4.48%, nitrogen (N): 1.32%, C/N: 3.39, pH: 7.09.

##### Etymology.

The specific epithet derives from *abbatissa*, Latin for abbess, as the species is dedicated to the abbess Emma, the first Abbess head of the Monastery of Sant Joan de les Abadesses, founded in 885 AC by her father, the Count of Barcelona, Guifré el Pilós. The Monastery was run by nuns until the year 1,017 when the female community was expelled, presumably for disorderly conduct, and replaced by monks.

##### Molecular characters.

**Figure 2.**
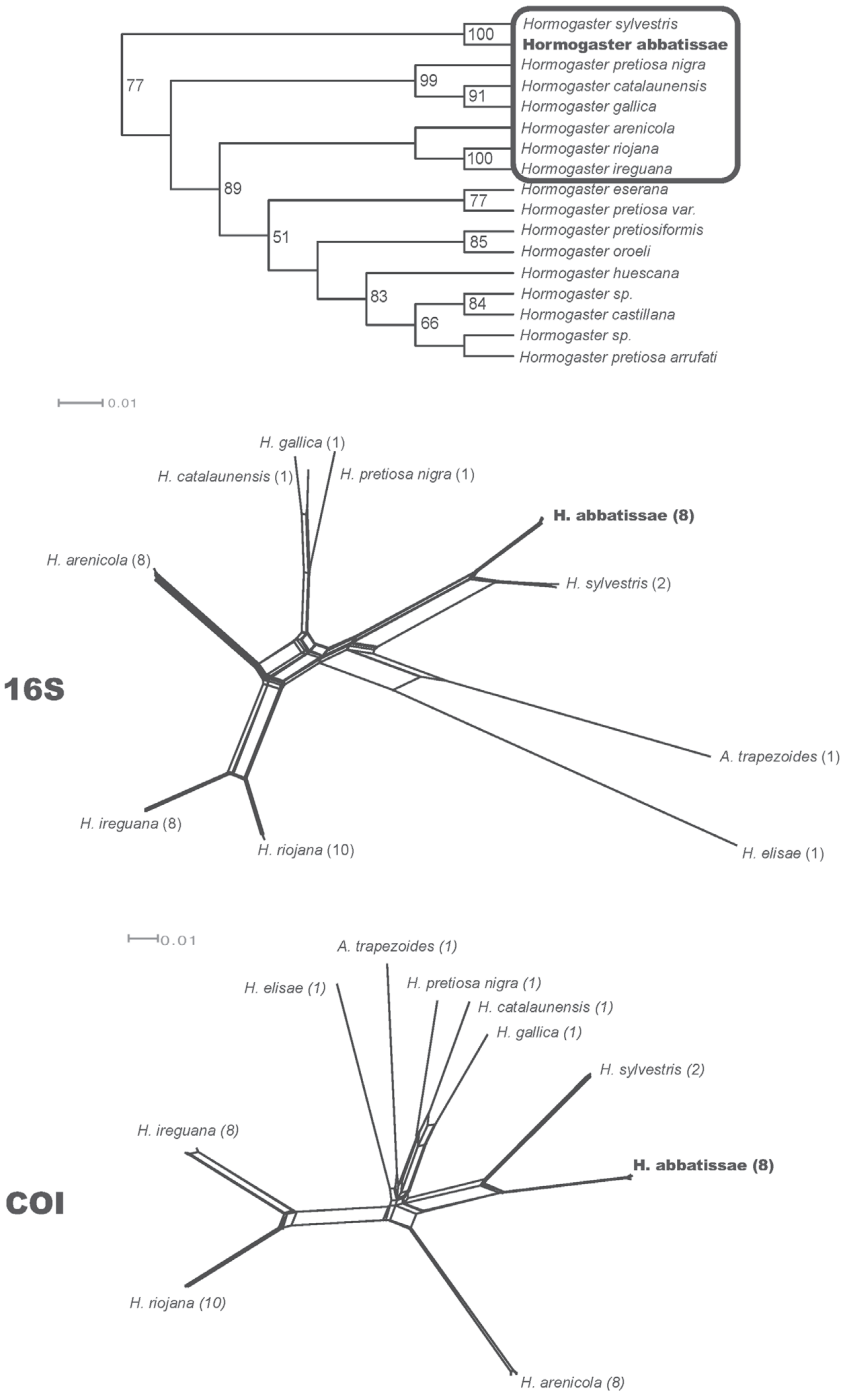
Top, part of the parsimony tree recovered by Novo et al. (2011), showing the clade where *Hormogaster abbatissae* was placed (in that work it is named *sp n*.). Bottom, network representation for 16S-tRNA and COI recovered by SplitsTree4 of the closest species (surrounded by a black square in the tree above) and *Hormogaster elisae* and *Aporrectodea trapezoides* as distant references.The number of specimens used is indicated in parenthesis.

Sequences from COI (8 individuals), 16S-tRNA (8 ind.), histone H3 (4 ind.), histone H4 (4 ind.), 28S rRNA (2 ind.) and 18S rRNA (1 ind.) were analysed with additional hormogastrid species. Phylogenetic analyses of the molecular data shows robust support for the monophyly of *Hormogaster abbatissae* sp. n., which is the sister species of *Hormogaster sylvestris* Qiu & Bouché, 1998 (Figure 2), described in the nearby locality of Montmajor (Barcelona, Spain). This clade forms the sister group to almost all other *Hormogaster* species from the NE Iberian Peninsula (see Novo et al. 2011 for details). This latter clade from the NE Iberian Peninsula splits into two groups, the first clade including *Hormogaster gallica* Rota, 1994 from Banyuls-sur-Mer (S of France), *Hormogaster catalaunensis* Qiu & Bouché, 1998 from El Brull (Barcelona, Spain) and *Hormogaster pretiosa nigra* Bouché, 1970 from Quillan (S of France). Its sister clade includes other *Hormogaster* species from the NE Iberian Peninsula, including *Hormogaster riojana* Qiu & Bouché, 1998 and related species (Figure 2).

Uncorrected pairwise distances for 16S-tRNA and COI are shown in Table 3 for the sister species *Hormogaster abbatissae* sp. n. and *Hormogaster sylvestris* and the morphologically-close *Hormogaster riojana* as well as its sister species *Hormogaster ireguana* Qiu & Bouché, 1998. *Hormogaster elisae* is included as a distant relative, even though it belongs to a possible new genus (see Novo et al. 2011).

The networks recovered by Splitstree4 for the COI and 16S genes including morphological and molecular closest species are shown in Figure 2.

GMYC analyses performed by Novo et al. (2012) identified *Hormogaster abbatissae*, *Hormogaster riojana* and *Hormogaster sylvestris* as different species.

##### Ecological characters. 

Soil characteristics in the localities where *Hormogaster abbatissae* sp. n., *Hormogaster riojana* and *Hormogaster sylvestris* occur are shown in Table 4. Differences in soil texture were detected: *Hormogaster sylvestris* and *Hormogaster riojana* inhabit Silt-loamy soils, whereas *Hormogaster abbatissae* sp. n. inhabits Clay-loamy soils. *Hormogaster abbatissae* sp. n. inhabits soils with a higher content in organic matter. Comparisons with the remaining species of the family were provided by Novo et al. (2012).

**Table 3.** Mean values of uncorrected pairwise differences in percentage obtained for 16S-tRNA (above the diagonal) and COI (below the diagonal, in bold) genes. Values of intraspecific differences are shown in the diagonal for the species that include more than one sequence type.

	*H. abbatissae*	*Hormogaster sylvestris*	*Hormogaster riojana*	*Hormogaster ireguana*	*Hormogaster elisae*
***H. abbatissae***	**0.10/**0.05	4.01	11.92	12.86	17.88
*Hormogaster sylvestris*	**11.71**	**0.46/**0.25	11.89	12.76	16.29
*Hormogaster riojana*	**17.80**	**17.36**	**0/**0.09	4.32	17.18
*Hormogaster ireguana*	**16.11**	**18.58**	**9.53**	**0.33/**0.03	17.72
*Hormogaster elisae*	**18.42**	**19.68**	**18.52**	**19.48**	**-**

**Table 4.** Soil characteristics in the sampling localities of *Hormogaster sylvestris* (Montmajor MAJ), *Hormogaster abbatissae* sp. n. (San Joan de les Abadesses, SAN) and *Hormogaster riojana* (Alesanco, ALE). CSand: coarse sand, FSand: fine sand, TSand: total sand, CSilt: coarse silt, FSilt: fine silt, Tsilt: total silt, Tex: textural class, SL: Silt loam, CL: Clay loam, C: percentage of carbon, N: percentage of nitrogen, C/N carbon/nitrogen relationship.

	**CSand**	**FSand**	**TSand**	**CSilt**	**FSilt**	**TSilt**	**Clay**	**Tex**	**C**	**N**	**C/N**	**pH**
**MAJ**	11.71	6.50	18.22	6.88	69.02	75.90	5.88	SL	2.98	0.83	3.6	7.39
**SAN**	13.57	9.62	23.18	6.27	32.37	38.64	38.18	CL	4.48	1.32	3.4	7.09
**ALE**	9.24	25.12	34.36	55.38	1.86	57.24	8.40	SL	1.63	0.30	5.33	7.33

## Discussion

Most species within the genus *Hormogaster* are very similar morphologically, with the clitellum, tubercula pubertatis, spermathecae and typhlosole, in addition to size or colour, being the key morphological characters traditionally used for species diagnosis. Table 5 includes a comparison of the characters of *Hormogaster abbatissae* sp. n. with those of its closest congeners, showing a large degree of overlap in the distribution of these characters and their states. In this case we have a species that appears the closest morphologically, *Hormogaster riojana*, collected in Alesanco, a locality ca. 420 km from Sant Joan de les Abadesses, that can be distinguished by the body and clitellum colour, shape of the tubercula pubertatis and the number of spermathecae (although *Hormogaster riojana* specimens with three pairs of spermathecae have been reported by Novo 2010). This could lead to consider *Hormogaster abbatissae* sp. n. a variety of *Hormogaster riojana*. Nevertheless, as shown by the phylogenetic and phylogeographic analyses of molecular data (see Figure 2), *Hormogaster riojana* appears distantly related to *Hormogaster abbatissae* sp. n.

The sister group of *Hormogaster abbatissae* sp. n. is *Hormogaster sylvestris* (Figure 2), collected in Montmajor, 50 km away from Sant Joan de les Abadesses. These two species, closely related phylogenetically and biogeographically, are easilydistinguished by their tubercula pubertatis (generally starting in more anterior segments and finer in *Hormogaster abbatissae* sp. n), clitellum (shorter and saddle shaped in *Hormogaster abbatissae* sp. n. and annular in *Hormogaster sylvestris*), spermathecae (three pairs in *Hormogaster abbatissae* sp. n. and two pairs in *Hormogaster sylvestris*) and typhlosole (15 lamellae in *Hormogaster abbatissae* sp n. and 13 in *Hormogaster sylvestris*). To these characters we can add other more variable characters such as colour, length, weight and number of segments (*Hormogaster sylvestris* is longer, heavier and with a higher number of segments). Of all these characters, the presence of three pairs of spermathecae in *Hormogaster abbatissae* sp. n. is the most conspicuous trait. It is therefore the combination of the morphological information and the phylogenetic position of the species, as derived from the molecular data, which aids in the global taxonomy of the group and serves to assess the degree of homoplasy in characters thought to be of taxonomic importance.

Some characters, such as the presence of Morren’s glands or the existence of a posterior gizzard, can be difficult to observe and of subjective interpretation. Morren’s glands seem to be absent because although an enrichment of blood vessels is detected in the oesophageal wall of some segments 10 – 14, the lamellae that define this organ were never observed. Likewise, the presence of a posterior gizzard is difficult to determine, as the gut thickens in segments 15 – 19 in the members of some species. However, in *Hormogaster abbatissae* sp. n. there is neither strong musculature, nor the thickening and hard covering of the lumen as observed in the gizzards of earthworms.

Regarding the molecular characters, Novo et al. (2009, 2010) proposed the presence of five cryptic species within the *Hormogaster elisae* complex, which resulted to be separated by genetic divergences between 9.41 – 18.31% for cytochrome *c* oxidase subunit I (Kimura 2-parameter distances, whose values are slightly higher than the uncorrected distances, used here). Also Hebert et al. (2003) reported comparable divergences for the same marker between 11.3%, for congeneric species of various animal groups and 15.7% between annelid species. It is evident, though that strict phenetic distances cannot be used for delimiting taxonomic boundaries, as other studies have shown that the same marker may have within species divergences much larger than the ones proposed by Hebert et al. (2003) (e.g., Barber et al. 2006, Boyer et al. 2007). This has been debated for earthworms by Chang and James (2011), who proposed that differences among species are indeed clade-specific, but they propose the existence of a consensus for COI (Kimura-corrected) distances: values under 9% normally indicate the same species, while values above 15% most probably indicate different species and values between 9-15% can be ambiguous. The species pairs *Hormogaster abbatissae* sp. n. and *Hormogaster sylvestris* as well as *Hormogaster riojana* and *Hormogaster ireguana* present COI uncorrected divergences within this ambiguity range (11.71% and 9.53%, respectively). The latest species were described by Qiu and Bouché (1998) based on morphology. Therefore, it seems that in this case distances need to be treated cautiously, thus reinforcing the critiques of their use for species delimitation (DeSalle et al. 2005, Hickerson et al. 2006, Whitworth et al. 2006). However, distances seem to be conservative in hormogastrid’s case and our data suggest that divergence below the level proposed by Chang and James (2011) may correspond to different species. Anyway it is clear the necessity of morphological data to verify the status of two lineages that present a divergence value within this range. Moreover, in the present case, these species are known to appear only in their type locality and therefore barcoding gap (ABGD) cannot be calculated with accuracy. Species delimitation with GMYC has been recently implemented in earthworms by Fernández et al. (2012) and particularly in hormogastrids by Novo et al. (2012). In both cases an overestimation of the species number, when compared with morphology, was detected. The marked genetic structure and scarce dispersion capacity of the studied earthworms could be the cause for this overestimation, being these factors particularly evident in *Hormogaster elisae*’s case, with various cryptic species. A GMYC analysis shows *Hormogaster abbatissae* sp. n. as a different entity from *Hormogaster sylvestris*. Whether the GMYC method is overestimating in this particular case is unknown but *Hormogaster abbatissae* sp. n., *Hormogaster riojana* and *Hormogaster sylvestris* are well-separated when combining morphology, phylogenetic analyses and network information.

After examining its morphology, phylogenetic placement and additional data such as GMYC and soil characteristics, it is evident that *Hormogaster abbatissae* sp. n. constitutes a new hormogastrid taxon not phylogenetically related to those species that show closest morphological similarities. Morphological and molecular data supply different signals thus clashing in the case of *Hormogaster abbatissae* sp. n. The question arising in this case is what should taxonomists do when different data sources provide conflict? The answer to this question is not straightforward. On the one hand, these animals can present a morphological stasis, as shown in *Hormogaster elisae* (Novo et al. 2009, 2010). On the other hand, molecular techniques rely on limited information, in this case based on a group of specific genes and depend on specific algorithms. This decision should thus be based on a profound knowledge of the morphological variability and peculiarities of the studied group, and an understanding of the strengths and weakness of the applied molecular analyses (used genes, sampling scheme, algorithms, etc.) that could lead to different decisions depending on the study case.

In this particular case, phylogeny is robust because it is based on a great amount of data, combining mitochondrial and nuclear genes (COI, 16S-tRNA, H3, H4, 28S, 18S) with different phylogenetic signal and including individuals representing most of the species in the family. Also we know that living conditions in the soil induce cryptic speciation processes in earthworms (King et al. 2008, Novo et al. 2009, 2010, James et al. 2010, Buckley et al. 2011, Dupont et al. 2011 – but see rebuttals of these “cryptic” genetic cladograms by Blakemore et al. 2010 and Blakemore 2010, 2011) and in many occasions the most important morphological characters used for earthworm species delimitations overlap showing a poor discrimination capacity (Fernández et al. 2012). Therefore, morphological characters should be applied cautiously by earthworm taxonomists in case of conflict with other data source.

Regarding ecological factors, some important differences are detected for texture and organic matter among soils of *Hormogaster abbatissae* sp. n., *Hormogaster sylvestris* and *Hormogaster riojana*. However, it should be considered that a single locality is known per species and that the discovery of other populations may show a higher ecological range.

In summary, this study evidences the need of complementing the morphological data with molecular characters data in taxonomy, especially in groups with limited morphological characters and rampant convergence in their functional morphology, perhaps due to strong selective pressure due to habitat restriction. This study also proves that in case of rather small genetic divergence (within the range of uncertainty), morphology can be also helpful to conclude complementing molecular sources. We propose to establish the new species *Hormogaster abbatissae* sp. n. Given the existence of species closely-related phylogenetically (*Hormogaster sylvestris*) and an unrelated but morphologically similar species (*Hormogaster riojana*), a more exhaustive sampling effort in NE Spain could provide new diversity to help evaluate this situation. As indicated by Sites and Crandall (1997), species descriptions are not facts, but hypothesis established when certain criteria available in a specific moment are fulfilled and they can be accepted or rejected when new data are available.

**Table 5.** Comparison of the morphological characters of *Hormogaster abbatissae* sp. n. with those in the morphologically closest species. N. segments: number of segments. N. typhlosole lamellae: number of typhlosole lamellae. Size, weight and number of segments are for adult specimens. For complete information of the rest of the species within Hormogastridae, see Qiu and Bouche (1998).

	***Hormogaster abbatissae***	***Hormogaster gallica***	***Hormogaster riojana***	***Hormogaster sylvestris***	***Hormogaster ireguana***
Colour	Grey-bluish	Dark brownish	Dark brownish	Colourless	Brownish-grey
Clitellum	14, 15–27 (28)Saddle shaped, beige	(13) 14–28 (29,30)*Saddle shaped	13,14, 17–27,28Saddle shaped, dark	15–28Annular	13–27Annular
Tubercula pubertatis	(20) 21,22–26,27Fine band	(22, 23) 24 – 27Fine and short band	(20)21–27Fine band	22–27Wide band	19–26Linear band
Intersetal ratio	50:1.5:9:1:52	69:1.3:8.8:1:66	55:1:13:1:65	50:2:10:1:50	120:1:20:1:100
Length	103–130	165–190	125–185	180–220	100
N. segments	239–270	250–433	243–278	350–420	223
Weight (g)	3.45–4.98	9.2–17		13.6–15.3	
Spermathecae(pores)Appearance	8,9,10(8/9,9/10,10/11)Simple, Multicameral	9, 10(9/10,10/11)Multiple, sessile, in a ring	9, 10(9/10,10/11)Simple,Multicameral	9, 10(9/10,10/11)Simple, Multicameral	8, 9, 10(8/9,9/10,10/11)Simple
N. typhlosole lamellae	15(2 very small)	13	15	13	19
Morren gland	Absent	Absent	Absent	Absent	Absent
Posterior gizzard	15? 16 17?Very weak	14–16?Weak	15–16Weak	16Weak	14–15Weak
Other characters		Carinated anterior segments			

## Supplementary Material

XML Treatment for Hormogaster abbatissae

## Appendix

Supplementary figure. (doi: 10.3996/zookeys.242.3996.app). File format: Adobe PDF file (pdf). **Explanation note:** Sampling area of *Hormogaster abbatissae* (A), alive specimen (B), fixed specimens (D) and their spermathecae from one side of the body (C) numbered from anterior to posterior.
