# Supplementary figures and images for: Compilation of morphological and molecular data, a necessity for taxonomy: The case of Hormogaster abbatissae sp. n. (Annelida, Clitellata, Hormogastridae)
Source: Zookeys. 2012 Nov 15;(242):1–16. doi: 10.3897/zookeys.242.3996 (PMC3560842; doi:10.3897/zookeys.242.3996)

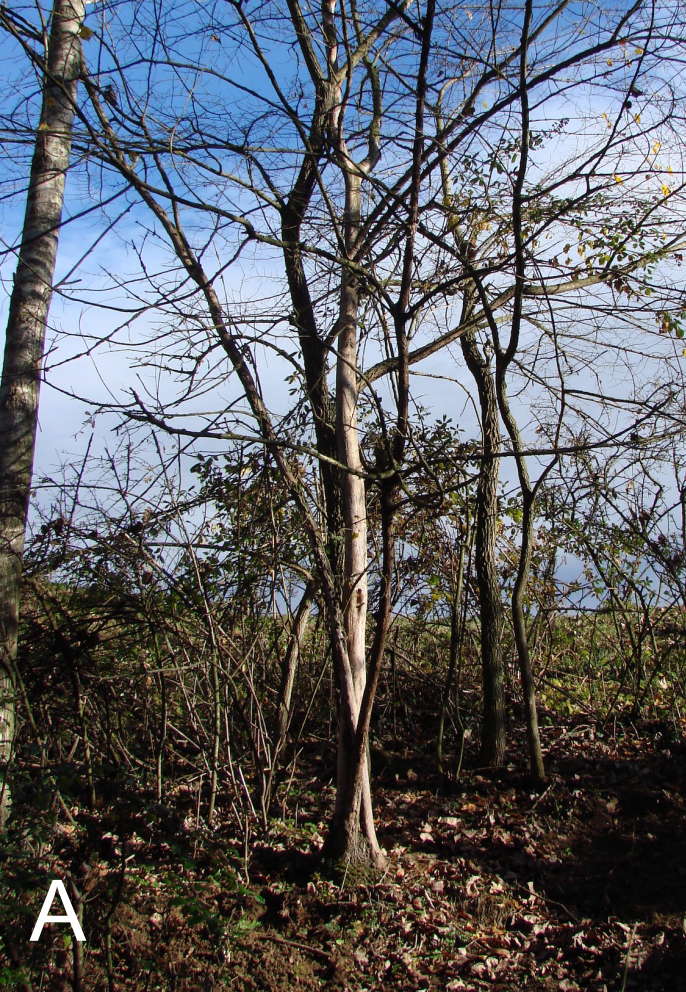

A

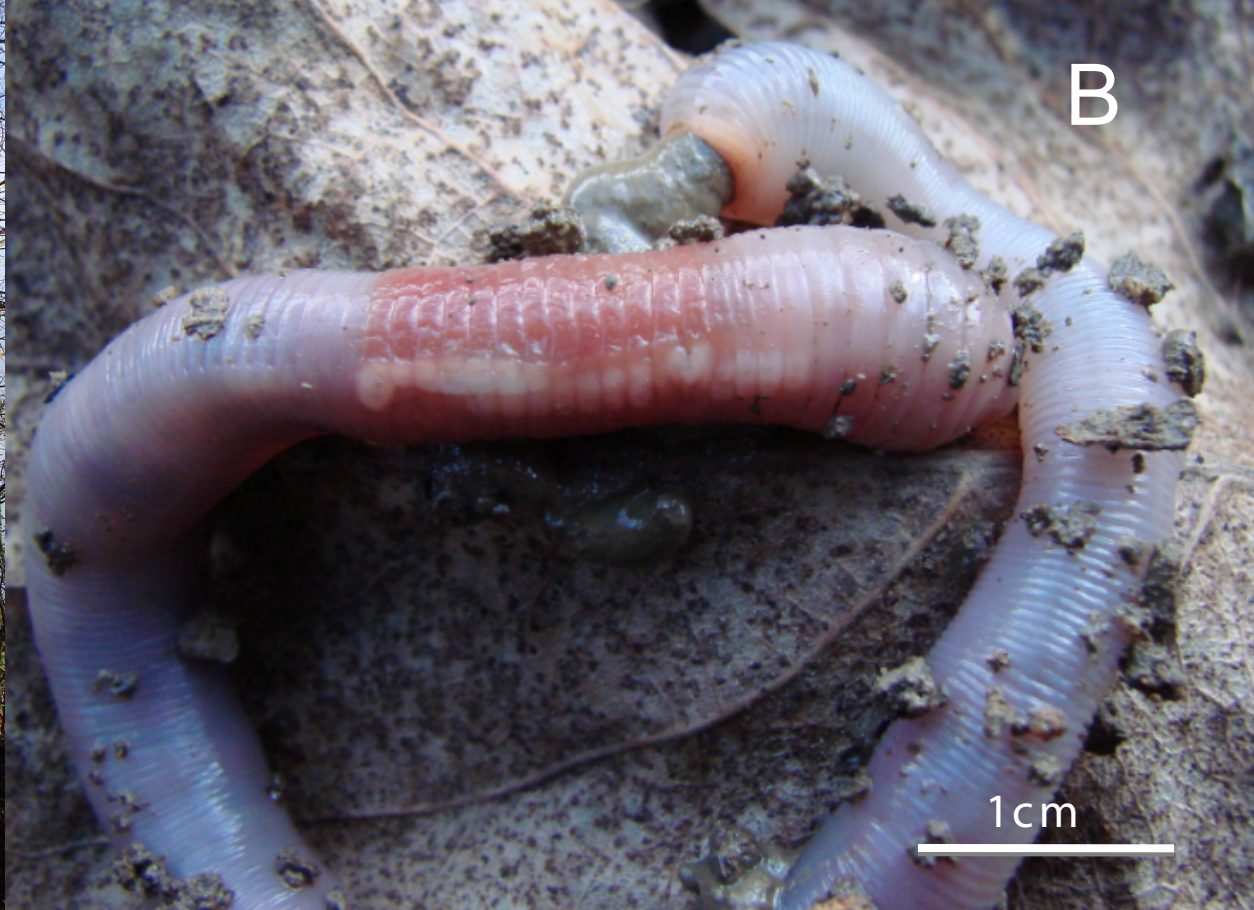

B

1cm

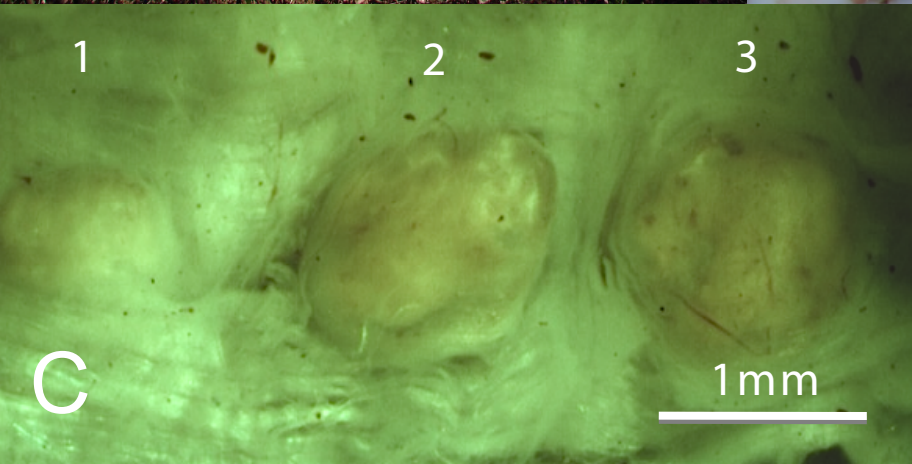

C

1mm

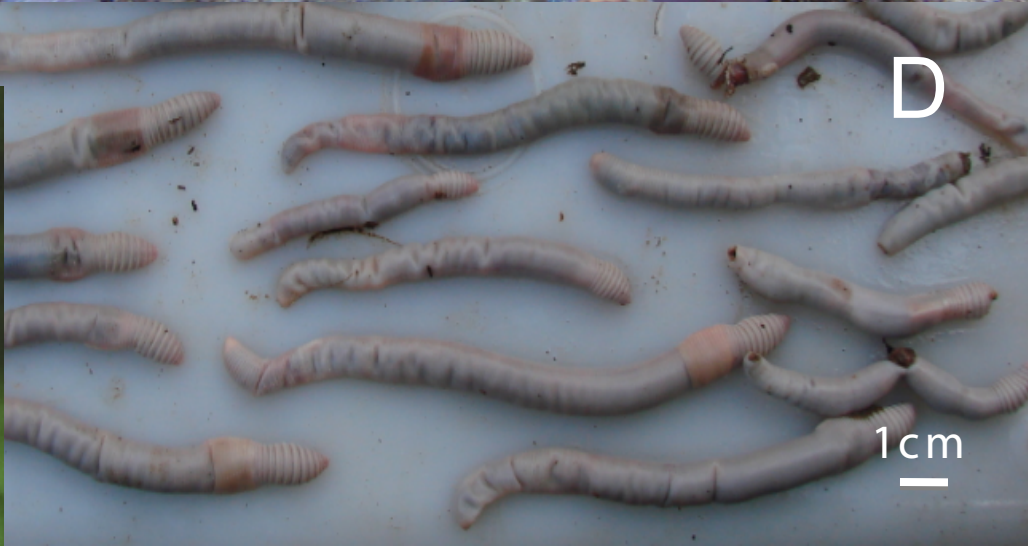

D

1cm

Supplement: Supplementary file 2 — Supplementary figure. (doi: 10.3996/zookeys.242.3996.app). File format: Adobe PDF file (pdf). Explanation note: Sampling area of Hormogaster abbatissae (A), alive specimen (B), fixed specimens (D) and their spermathecae from one side of the body (C) numbered from anterior to posterior. [file ZooKeys-242-001-s001.pdf]
